# A Validation Study of Methylated Syndecan-2 in Stool DNA for the Detection of Colorectal Cancer

**DOI:** 10.3390/diagnostics16121901

**Published:** 2026-06-19

**Authors:** Hyoung Il Choi, Yoon Dae Han, Jae Myung Cha, Nam Kyu Kim

**Affiliations:** 1 Department of Internal Medicine, Kyung Hee University Hospital at Gangdong, College of Medicine, Kyung Hee University, Seoul 05278, Republic of Korea; fullhouse11245@naver.com; 2 Division of Colorectal Surgery, Department of Surgery, Severance Hospital, College of Medicine, Yonsei University, Seoul 03722, Republic of Korea; acylyoon@yuhs.ac; 3 Division of Colorectal Surgery, Department of Surgery, Yongin Severance Hospital, Seoul 16995, Republic of Korea; namkyuk@yuhs.ac

**Keywords:** biomarkers, colorectal neoplasm, DNA methylation, fecal occult blood test, mass screening

## Abstract

**Background/Objectives**: The stool DNA-based SDC2 methylation (meSDC2) test has emerged as a promising noninvasive tool for the early detection of colorectal cancer (CRC). This study aimed to validate the clinical performance of the meSDC2 test for CRC detection in a multicenter hospital setting. **Materials and Methods**: This prospective, retrospective, multicenter, single-blind, case–control study was conducted at three tertiary medical centers. The primary endpoints were sensitivity and specificity for CRC detection. Secondary endpoints included test performance by tumor stage and location, and positivity rates in non-CRC lesions. **Results**: Among 636 participants, 260 (40.9%) had CRC, 173 (27.2%) had colorectal polyps, and 182 (28.6%) had normal colonoscopy findings. The meSDC2 test demonstrated a sensitivity of 87.7% (95% CI: 83.7–91.7%) and a specificity of 86.2% (95% CI: 82.7–89.7%) for CRC detection, with an AUC of 0.869 (95% CI: 0.841–0.895). Specificity among participants with negative colonoscopy findings was 91.8%. Positivity rates were 28.3% (95% CI: 15.3–41.3%) for advanced adenomas and 13.8% (95% CI: 6.6–21.0%) for non-advanced adenomas. Compared with the fecal immunochemical test (FIT), the meSDC2 test showed significantly higher sensitivity, whereas FIT demonstrated higher specificity. Combined testing improved sensitivity to 95.6% and 82.4% specificity. The meSDC2 test also showed significantly greater sensitivity than FIT for early-stage CRC (*p* = 0.037), while combined testing further improved sensitivity for both early- and late-stage CRC(*p* = 0.037 and *p* = 0.006, respectively). **Conclusions**: The stool-based meSDC2 test demonstrated high sensitivity, specificity, and consistent diagnostic performance across subgroups, supporting its clinical utility as a reliable and noninvasive CRC screening tool.

## 1. Introduction

Colorectal cancer (CRC) is the third most common cancer worldwide and the second leading cause of cancer-related mortality [1]. Recently, the incidence of CRC has been rapidly increasing in many Asian countries, including Republic of Korea [2]. Because most CRCs develop through the adenoma–carcinoma sequence over 5–10 years, early detection of precursor lesions and early-stage cancers offers a critical opportunity for timely therapeutic intervention and improved survival outcomes [3,4]. To effectively prevent CRC, screening through organized, population-based programs should be promoted. In Korea, the National Cancer Screening Program provides an annual fecal immunochemical test (FIT) for average-risk adults aged ≥50 years [5]. However, FIT has relatively low sensitivity, detecting approximately 70% of early-stage CRCs and only approximately 20% of advanced adenomas (AAs), which restricts its effectiveness as a stand-alone screening tool [6,7]. In addition, colonoscopy—although the gold standard—remains invasive and suboptimal as a primary screening tool due to the need for bowel preparation, procedural discomfort, sedation-related risks, and potential complications.

Accordingly, novel noninvasive methods are urgently required for the early detection of CRC using emerging biomarkers. In the United States, the multitarget stool DNA test (Cologuard™, Exact Science Corporation, WI, USA) has been commercialized as a noninvasive screening modality that combines FIT with stool-derived DNA biomarkers [8]. This test has been approved by the Food and Drug Administration (FDA), demonstrating a sensitivity of 92% and a specificity of 87% for CRC detection. In Korea, a stool DNA-based Syndecan-2 (SDC2) methylation (meSDC2) test has also been approved for commercial use owing to its high sensitivity and specificity for CRC as EarlyTect^®^ Colon Cancer (Genomictree Inc., Daejeon, Republic of Korea) [9,10]. Because hypermethylation of CpG islands within gene promoter regions is a common epigenetic event in colorectal carcinogenesis, stool DNA methylation-based tests may further enhance CRC detection [11,12]. Although previous pivotal studies reported high sensitivity and specificity of the stool DNA-based me*SDC2* assay, post-marketing validation under real-world clinical conditions remains limited. Following its commercial launch, it is essential to verify whether the clinical performance of the marketed product is consistent with that observed in prior clinical studies [9]. In the present study, we evaluated the diagnostic performance, reproducibility, and practical utility of the me*SDC2* test for detecting colorectal neoplasia (CRN) in a multicenter setting.

## 2. Materials and Methods

### 2.1. Study Design

This Post-Market Surveillance (PMS) study employed a prospective, retrospective, multicenter, single-blinded, case–control design to evaluate the clinical performance of the stool DNA-based *SDC2* methylation test for CRC detection. This study was conducted between December 2022 and May 2025 at three medical institutions in Korea.

**Figure 1:**
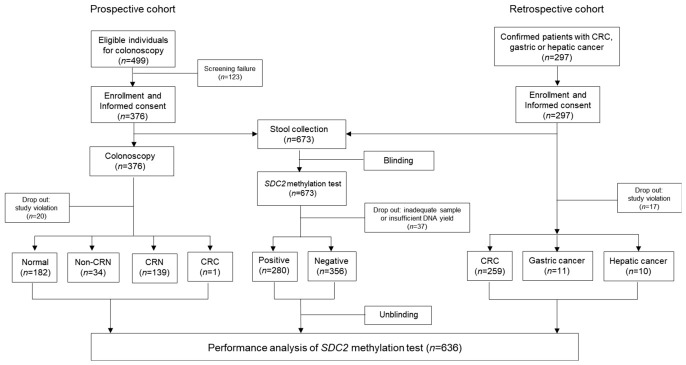
Flow chart of the study design. A prospective and retrospective, multicenter, single-blinded, case–control study to evaluate the clinical performance of a stool DNA-based *SDC2* methylation test. CRN, colorectal neoplasia; CRC, colorectal cancer.

The prospective cohort included individuals scheduled to undergo colonoscopy for routine CRC screening, positive FIT results, gastrointestinal symptoms, or post-polypectomy surveillance. The retrospective cohort comprised patients with newly diagnosed CRC, gastric cancer or liver cancer during the study period (Figure 1). Because of the relatively low prevalence of CRC in the general population, patients newly diagnosed with CRC from both the prospective and retrospective cohorts were included in the CRC (case) group to ensure sufficient statistical power for sensitivity analysis. The control group included individuals with normal colonoscopy findings, defined as no CRC or colorectal polyps. The reference group comprised patients with colorectal polyps identified in the prospective cohort, as well as patients with gastric or liver cancer from the retrospective cohort, to further assess test specificity.

All eligible participants submitted their fecal samples for both the *SDC2* methylation test and FIT. Sample identifiers were anonymized and laboratory investigators remained blinded to sample identity (single-blind design) through testing. Fecal samples were transported to a designated testing laboratory where the investigational device test was performed. After all testing was completed, blinding was lifted, and test results were compared with colonoscopic, pathological, and radiological findings, which serve as the reference standards.

Quantitative FIT was performed using the OC-Sensor DIANA Micro desktop analyzer (Eiken Chemical Co., Tokyo, Japan). According to the manufacturer’s instructions, a cutoff value of 20 μg Hb/g feces (100 ng Hb/mL buffer) was used to define FIT positivity. This study was approved by the Institutional Review Board of Kyung Hee University Hospital at Gangdong, Seoul, Republic of Korea (IRB No. PMS 2022-027) and by the institutional review boards of all participating institutions. Written informed consent was obtained from each participant prior to enrollment in the study.

### 2.2. Participants

Adults aged 30–80 years who provided written informed consent were enrolled in this study. The age criteria were aligned with the labeling requirements of the Ministry of Food and Drug Safety of Korea for the *SDC2* methylation test. Patients with CRC or gastric or hepatic cancer in the retrospective cohort were eligible if their diagnoses had been confirmed by pathological or radiological examination and they had not received any anticancer treatment, including surgery, chemotherapy, or radiotherapy. Fecal specimens were obtained from eligible participants and were required to be submitted within 4 weeks of collection. Only non-diarrheal (formed) stool samples with a net fecal weight of ≥2 g were acceptable for analysis.

Participants were excluded if they declined participation, had a previous history of CRC, had undergone prior anticancer treatment for CRC, gastric cancer, or liver cancer, or had incomplete clinical information. Participants were also excluded from the final analysis if stool samples were inadequate for testing, including samples with less than 2 g of stool, samples submitted more than 4 weeks after collection, diarrheal samples, or samples collected during bowel preparation. The full analysis set was defined as participants who underwent the *SDC2* methylation test and completed at least one efficacy assessment. The per-protocol set included participants in the full analysis set who completed the clinical performance evaluation without any major protocol deviations. In this study, the full analysis and per-protocol sets comprised the same participants.

### 2.3. Data Collection

Clinical data were collected using standardized case report forms and included: institutional and physician data; patient demographics (age and sex); *meSDC2* test results; FIT results; and colonoscopy findings, including lesion size, location, number, and histopathological diagnosis. Based on complete colonoscopy and pathological evaluations, participants were categorized as normal (including those with non-specific lesions), colorectal polyps, or CRC. Colorectal polyps were further classified as CRN—including AA, non-AA, and serrated lesions—or non-CRN, which included hyperplastic and inflammatory polyps.

Lesion size was determined using pathological measurements and endoscopic estimates. Right-sided lesions were defined as those located at or proximal to the splenic flexure, whereas left-sided lesions were defined as those distal to the splenic flexure. For participants with multiple CRN lesions, classification was based on the most advanced histological lesion or the largest lesion. When the initial diagnostic colonoscopy was incomplete or inadequate, only data from subsequent adequate colonoscopies were included in the final analysis.

### 2.4. Stool DNA-Based SDC2 Methylation Test

The stool collection procedure followed previously established protocols [9,10]. Briefly, at least 2 g of stool was collected from four to five different sites using a stool collection kit (Genomictree, Inc., Daejeon, Republic of Korea). All samples were obtained before any therapeutic intervention or colonoscopy and were immediately transferred to a central laboratory for methylation analysis [9]. Genomic DNA was extracted from stool specimens using the solid-phase, magnetic bead-based GT Nucleic Acid PREP Kit II (Genomictree, Inc.) according to the manufacturer’s protocol. DNA concentration was quantified using the Qubit dsDNA BR Assay Kit (Thermo Fisher Scientific, Waltham, MA, USA). A total of 2.0 μg of purified DNA was subjected to sodium bisulfite modification using the EZ DNA Methylation-Gold kit (Zymo Research, Irvine, CA, USA) following the manufacturer’s instructions. Bisulfite-converted DNA was used immediately for methylation analysis or stored at −20 °C until methylation analysis. Stool samples were collected using a dedicated collection kit containing a DNA stabilization buffer (Genomictree, Inc., Daejeon, Republic of Korea). Specimen stability was evaluated using three me*SDC2*-negative and three me*SDC2*-positive stool samples, each tested in triplicate. Analytical validation confirmed that stool specimens remained stable for up to 30 days at room temperature [10]. Detailed stability data are provided in Supplementary Table S2.

The *SDC2* gene methylation was assessed using the EarlyTect^®^ Colon Cancer assay (Genomictree, Inc.) on an AB 7500 Fast Real-Time PCR system (Thermo Fisher Scientific), with two replicate PCR reactions performed per sample [10]. An *SDC2*-positive result was defined as a C_T_ value < 40 in at least one of the two replicate reactions (1/2 interpretive algorithm). Samples with no amplification in either reaction were classified as negative. Assay validity required a *COL2A1* C_T_ value < 33 in both reactions; otherwise, the DNA was re-extracted from the remaining stool specimen and retested. The cutoff value for CRC detection was prespecified [10]. For samples in which *SDC2* methylation was not detected, the C_T_ (cycle threshold) value was assigned the maximum cutoff of 40 and converted to a value of 40–C_T_, yielding a methylation value of 0.0 for undetected samples.

### 2.5. Statistical Analysis

The primary endpoints were the diagnostic sensitivity and specificity of the me*SDC2* test for CRC detection. Secondary endpoints included sensitivity according to CRC stage and tumor location, as well as positivity rates in patients with non-CRC colorectal lesions, gastric cancer, and liver cancer. Sample size determination was based on the same rationale used in a previous clinical validation study of the stool DNA-based me*SDC2* assay [9], in which the assay demonstrated an overall sensitivity of 90.2% and specificity of 90.2% for CRC detection. The present study was designed to confirm whether the sensitivity exceeded 70% and the specificity exceeded 80%. Assuming a one-sided type I error rate of 0.025 and a statistical power of at least 90%, a minimum of 241 patients with CRC and 241 subjects with negative colonoscopic findings were required.

Given that the estimated prevalence of CRC in the general screening population is approximately 0.5% [8], a prospective-only design would not have provided a sufficient number of CRC cases for reliable sensitivity analysis. Therefore, 260 patients with histologically confirmed CRC were retrospectively enrolled to ensure adequate case numbers. In addition, 330 participants were prospectively recruited prior to colonoscopic diagnosis to reflect real-world screening conditions. Considering an anticipated dropout rate of approximately 10%, the final target sample size was set at 656 participants. To further assess specificity, patients with gastric cancer and liver cancer were additionally included. Diagnostic sensitivity, specificity, positive predictive value (PPV), negative predictive value (NPV), and area under the receiver operating characteristic curve (AUC) were calculated using a predefined cutoff value, along with their corresponding 95% confidence intervals (CIs). Statistical significance was defined as a two-sided *p*-value of <0.05.

Continuous variables were presented as mean ± standard deviation (SD) and compared using Student’s *t*-test. Categorical variables were expressed as numbers (percentages) and compared between subgroups using Fisher’s exact test or *χ*^2^ tests. All statistical tests were two-sided, and a *p*-value < 0.05 was considered statistically significant. Statistical analyses were performed using MedCalc V22.023 (MedCalc Software Ltd., Ostend, Belgium).

## 3. Results

A total of 796 participants were screened, of whom 123 were excluded (Figure 1). Reasons for exclusion were violation of the inclusion criteria (*n* = 3), withdrawal of consent (*n* = 6), colonoscopy not performed (*n* = 9), failure to submit a stool sample (*n* = 69) and participants with insufficient (<2 g; *n* = 36) stool weight. 673 participants were initially enrolled. An additional 37 samples were excluded during testing for the following reasons: inability to classify colonoscopy findings into predefined groups (*n* = 5), inappropriate sample storage or insufficient DNA yield (*n* = 24), failure to meet sample adequacy criteria (*n* = 4), and suboptimal colonoscopy (*n* = 4). In total, 636 samples were included in the final analysis.

### 3.1. Characteristics of the Study Population

The demographic characteristics and distribution of disease types among the study participants are presented in Table 1. Among the 636 participants in the efficacy analysis set, 260 (40.9%) had CRC, 173 (27.2%) had colorectal polyps, and 182 (28.6%) were confirmed as normal by colonoscopy. In addition, 11 patients (1.7%) had gastric cancer and 10 (1.6%) had liver cancer. Of the 260 patients with CRC, 68 (26.1%) had right-sided CRC, 191 (73.5%) had left-sided CRC, and one (0.4%) had tumors involving both the right and left colon. The distribution of CRC stages was as follows: stage 0–I, 64 patients (24.6%); stage II, 56 patients (21.5%); stage III, 94 patients (36.2%); and stage IV, 46 patients (17.7%). Among the 173 colorectal polyps, 139 (80.3%) were classified as CRN, while 34 (19.7%) were classified as non-CRN.

### 3.2. The meSDC2 Test Results

**Figure 2:**
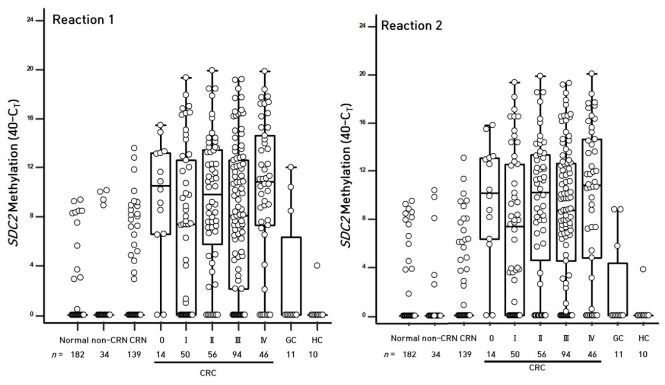
Results of the *meSDC2* test from two reactions in stool DNA expressed as C_T_ values representing the methylation level of the SDC2 gene. CRN, colorectal neoplasia; CRC, colorectal cancer; GC, gastric cancer; HC, hepatic cancer.

The me*SDC2* test results for the study population are presented as C_T_ values, which reflect the SDC2 gene methylation level (Figure 2). Most participants confirmed as normal by colonoscopy exhibited little to no detectable methylation. In contrast, patients with CRC demonstrated a high frequency of methylation positivity, regardless of disease stage. Methylation was also detected among individuals with CRN (excluding CRC), although at a lower frequency than in those with CRC. A small number of patients with gastric or liver cancers also showed SDC2 methylation, but at markedly lower frequencies compared with CRC cases.

### 3.3. Diagnostic Performance of the meSDC2 Test for CRC

A total of 260 patients with CRC were compared with 376 non-CRC controls, including colonoscopy-negative individuals and patients with colorectal polyps, gastric cancer or liver cancer, corresponding to a false-negative rate of 12.3% and a false-positive rate of 13.8% (Table 2). The me*SDC2* test demonstrated a sensitivity of 87.7% (95% CI: 83.7–91.7%) and a specificity of 86.2% (95% CI: 84.2–89.4%), with an AUC of 0.869 (95% CI: 0.841–0.895) for CRC detection. The overall diagnostic accuracy was 86.2% (95% CI: 84.2–89.4%). Specificity among participants with negative colonoscopy findings was 91.8% (95% CI: 87.8–95.8%). The PPV was 3.1% (95% CI: 2.4–4.0%). The NPV was 99.9%, indicating excellent rule-out performance for CRC. Of the 260 CRC cases included in this study, 259 were enrolled retrospectively, whereas only one was identified within the prospective colonoscopy-scheduled cohort.

The clinical performance of the me*SDC2* test did not differ significantly according to age, sex, or testing year (Table 3). Sensitivity across age groups ranged from 80.0% in participants aged 30–39 years to 92.0% in those aged 40–49 years (*p* = 0.861). Specificity across the age groups ranged from 89.6% to 100.0% (*p* = 0.832). No significant differences were observed between males and females in sensitivity (87.4% vs. 88.0%; *p* = 0.885) or specificity (91.5% vs. 91.9%; *p* = 0.935). Similarly, test performance was consistent across testing years, with comparable sensitivity (88.8% vs. 87.1%; *p* = 0.705) and specificity (92.4% vs. 89.5%; *p* = 0.566) in 2023 and 2024, respectively.

### 3.4. Positive Rate of Test for CRC According to Location and Stages

The positivity rate of the me*SDC2* test did not differ significantly according to tumor location or TNM stage (Table 4). By location, positivity was 86.8% (59/68; 95% CI: 78.7–94.8%) in right-sided CRCs and 88.0% (168/191; 95% CI: 83.3–92.6%) in left-sided CRCs (*p* = 0.798). Across individual colorectal segments, positivity rates ranged from 60.0% to 100.0% (*p* = 0.186). Positivity by TNM stage varied from 85.7% in stage 0 to 91.3% in stage IV (*p* = 0.671), with no statistically significant differences observed across stages.

### 3.5. Comparative Analysis of the Clinical Performance

Among the 617 participants who underwent both the me*SDC2* test and FIT (Table 5), the sensitivity of the me*SDC2* test was significantly higher than that of the FIT (87.1% vs. 81.0%; *p* = 0.045) (*n* = 248), whereas FIT demonstrated higher specificity (94.3% vs. 86.2%; *p* < 0.001) (*n* = 369). When both tests were combined, the clinical sensitivity and specificity for detecting CRC increased to 95.6% (95% CI: 93.0–98.1%) and 82.4% (95% CI: 78.5–86.3%), respectively. Compared with the me*SDC2* test alone, the combined test demonstrated significantly higher sensitivity (95.6% vs. 87.1%; *p* < 0.001) but no significant difference in specificity (86.2% vs. 82.4%; *p* = 0.188). When sensitivity was compared by TNM stage, the *SDC2* methylation test showed significantly higher sensitivity than FIT for early-stage (0–II) CRCs (84.2% vs. 72.8%; *p* = 0.037), whereas no significant difference was observed for late-stage (III–IV) CRCs (*p* = 0.741). Compared with the *SDC2* methylation test alone, the combined test showed improved sensitivity for both early- and late-stage CRC (*p* = 0.037 and *p* = 0.006, respectively).

### 3.6. Positive Rate of Test for Non-CRC Lesions

The positivity rates of the *SDC2* methylation test for non-CRC lesions are presented in Supplementary Table S1. The overall positivity rate for CRN was 18.7% (26/139; 95% CI: 12.2–25.2%). In the subgroup analysis, the positivity rate was 28.3% (13/46; 95% CI: 15.3–41.3%) for AA, 13.8% (12/87; 95% CI: 6.6–21.0%) for non-AA, and 16.7% (1/6; 95% CI: 0.0–46.5%) for serrated lesions. The overall positivity rate for non-CRN was 17.6% (6/34; 95% CI: 4.8–30.5%). Among patients with gastric cancer, the positivity rate was 27.3% (3/11; 95% CI: 1.0–53.6%), whereas among those with hepatic cancer, it was 20.0% (2/10; 95% CI: 0.0–44.8%).

## 4. Discussion

Previous studies have identified *SDC2* methylation as a sensitive biomarker for the early detection of CRC using stool DNA [9,10,12]. However, additional validation of the clinical performance of the *SDC2* methylation test for CRC is warranted. To the best of our knowledge, this is the first validation study of a commercially available stool DNA-based *SDC2* methylation test, and it is also the first to compare its clinical performance with that of FIT for CRC detection. In this study, the clinical sensitivity of the *SDC2* methylation test for CRC was 87.7% (95% CI: 83.7–91.7%), and the specificity was 86.2% with an AUC of 0.869, indicating high diagnostic accuracy. The clinical performance targets of ≥70% sensitivity and ≥80% specificity—established in earlier clinical trials for regulatory approval [9]—were reaffirmed as the benchmarks in the present study and were significantly exceeded (*p* < 0.01), demonstrating robust diagnostic performance under real-world conditions. In the pivotal clinical trial conducted for regulatory approval, the test revealed a sensitivity of 90.2% and a specificity of 90.2% [9]. Comparative analyses between the pivotal trial for regulatory approval [9] and the current PMS study showed reproducible clinical performance, with no statistically significant differences in sensitivity (*p* = 0.369) or specificity (*p* = 0.582). These findings highlight the consistent diagnostic performance of the *SDC2* methylation test from the approval phase through post-marketing clinical use, supporting both its clinical validity and high reproducibility. Moreover, no significant differences in sensitivity or specificity were observed across subgroups stratified by sex, age, and year of testing. Sensitivity also did not differ significantly according to CRC stage or tumor location. These findings demonstrate that the *SDC2* methylation test maintains consistent and reliable performance across diverse demographic and clinical contexts. The analytical performance of the meSDC2 stool assay has been previously characterized by Kim et al. [10]. The assay demonstrated a limit of detection of 36.9 pg of me*SDC2* DNA, equivalent to approximately 10 diploid genome copies. In the limit of blank evaluation using me*SDC2*-negative stool DNA samples, no false-positive results were observed (40/40 negative agreement), confirming negligible background amplification and high analytical specificity.

A key finding of this study is the exceptionally high NPV (99.9%), which indicates that the test can effectively rule out CRC in individuals without CRC—with important implications for reducing unnecessary colonoscopies, alleviating patient burden, and lowering healthcare costs. The test may be particularly useful for significantly older patients or those with a history of abdominal surgery, for whom colonoscopy-related complications are a concern. FDA–approved products for CRC screening, such as multitarget stool DNA test (Cologuard™) and multitarget stool RNA test (ColoSense™), have also demonstrated high NPVs, supporting their effectiveness as noninvasive screening tools. The effectiveness of both tests is largely attributable to their high NPV; the multitarget stool DNA test demonstrated an NPV of 99.9% [8] and the multitarget stool RNA test showed an NPV of 99.96% [13]. In the present study, the NPV of the *SDC2* methylation test was comparable to those of FDA–approved products, suggesting its potential as a promising tool for CRC differential diagnosis. Additionally, the PPV of this test (3.1%) was 4.5 times lower than the 14.1% reported for the multitarget stool DNA testing [8], indicating it may reduce false-positive results and thereby improve overall screening efficiency. In the Korean National CRC Screening Program, the pooled PPV of the FIT for CRC detection over 14 years was only 2.1% [14], which is substantially lower than the PPV observed in this study. These findings suggest that the current test may offer a more efficient CRC screening option.

Among the 173 participants with identified colorectal polyps, the *SDC2* methylation test demonstrated a positivity rate of 18.7% (95% CI: 12.2–25.2%) for CRN and 17.6% (95% CI: 4.8–30.5%) for non-CRN. These rates were markedly lower than those observed in patients with CRC, suggesting that the *SDC2* methylation test exhibits high disease specificity for CRC. The lower sensitivity for premalignant lesions may be attributable to the smaller number of exfoliated tumor cells shed into the stool from precancerous lesions compared with invasive CRC [9,15,16,17,18]. In contrast, the sensitivity for stage 0 CRC was 85.7%, indicating that the test can effectively identify CRC at a very early stage. Accumulating evidence suggests that *SDC2* hypermethylation occurs early in colorectal carcinogenesis and increases in frequency progressively along the adenoma-carcinoma sequence [9,10,11,16,17,18]. *SDC2* methylation therefore represents an early epigenetic alteration amenable to stool-based detection of colorectal neoplasia, rather than a molecular change confined to advanced-stage disease. In this study, 32 of the 260 patients with CRC were not detected by the test, corresponding to a false-negative rate of 12.3%. This rate may reflect tumor cell shedding below the detection limit of the assay. Strategies such as testing the entire stool sample, employing multitarget stool DNA methods, or performing repeated stool collections may reduce false-negative results. However, these approaches increase analytical complexity, incur higher costs, and reduce scalability. Therefore, colonoscopy should still be considered when clinical suspicion for CRC remains high, even in the presence of a negative test result. Conversely, 52 of the 376 non-CRC control participants showed positive test results, corresponding to a false-positive rate of 13.8%. These false-positive results may arise from non-specific nucleic acid amplification associated with heterogeneous stool samples or missed CRN during a colonoscopy.

Because few prior studies have directly compared the *SDC2* methylation test with the FIT [19], this study is unique in providing a head-to-head comparison of these two screening modalities. When directly compared, the *SDC2* methylation test demonstrated significantly higher sensitivity than the FIT, whereas the FIT exhibited higher specificity. When the two tests were combined, the clinical sensitivity and specificity for CRC detection increased to 95.6% and 91.1%, respectively. In a previous Chinese study [19], the sensitivity for CRC (*n* = 111) increased to 97.3% when the *SDC2* methylation test was combined with the FIT, compared with 79.3% for the *SDC2* methylation test alone and 93.7% for FIT alone—findings consistent with the results of the present study. These results suggest that the two tests are complementary and that their combined use should be strongly considered for CRC screening. When sensitivity was analyzed according to TNM stage, the *SDC2* methylation test showed significantly higher sensitivity than the FIT for early-stage CRC (*p* = 0.037). Compared with the *SDC2* methylation test alone, the combined test demonstrated improved sensitivity for both early- and late-stage CRC (*p* = 0.037 and *p* = 0.006, respectively). As previous studies have reported lower positivity rates and pooled PPVs for the FIT than for the *SDC2* methylation test [14,20,21], the combined test may offer advantages over each test alone for CRC screening. The potential benefit of combining FIT and me*SDC2* testing derives from the complementary biological information provided by the two assays. While FIT detects bleeding-associated lesions, me*SDC2* reflects tumor-specific epigenetic alterations. Because both tests can be performed using stool specimens, patient burden remains minimal. However, the additional laboratory complexity and costs associated with dual-assay screening warrant further evaluation through prospective implementation and cost-effectiveness studies.

Aberrant *SDC2* methylation has been reported to be associated with the Lauren classification subtype during early gastric tumorigenesis [22], and detectable stool DNA *SDC2* methylation was also reported in patients with gastric cancer and liver cancer [9]. Based on these findings, we included patients with gastric and liver cancers as disease controls rather than using only normal colonoscopy subjects. The rationale for this approach was to evaluate the CRC specificity of stool-based SDC2 methylation testing under more clinically relevant conditions and to assess potential cross-reactivity in non-colorectal gastrointestinal malignancies. In the present study, me*SDC2* positivity was observed in 27.3% of gastric cancer patients and 20.0% of liver cancer patients, which was consistent with previously reported rates. These positivity rates were substantially lower than those observed in CRC, supporting that false positivity due to these cancers is relatively limited and that the assay maintains high specificity for CRC detection. Analytical quality control is also essential for the reliable implementation of stool DNA methylation testing in clinical practice. The me*SDC2* assay demonstrated excellent analytical precision, with coefficients of variation below 5% for repeatability, inter-site reproducibility, and lot-to-lot variation [10]. To minimize variability arising from differences in technicians, instruments, and reagent lots, standardized operating procedures, validated PCR instrumentation, centralized laboratory testing, predefined interpretation algorithms, and comprehensive run controls were employed throughout the study. Collectively, these measures support the robustness and reproducibility of the assay across diverse testing conditions and clinical settings.

This study has some limitations. First, it employed a combined retrospective and prospective design due to the low prevalence of CRC in the prospective cohort. This approach was selected to account for (1) the relatively small number of CRC cases in the prospective cohort, which could limit the statistical power for assessing test performance, and (2) the fact that individuals with negative test results in real-world practice typically did not undergo colonoscopy, preventing the accurate calculation of sensitivity and specificity. The present study was designed as a cross-sectional diagnostic accuracy study and, therefore, long-term outcome data—including interval cancer incidence following negative results and the clinical significance of false-positive findings—were not captured; future prospective studies incorporating longitudinal follow-up are warranted. Second, as this study was conducted in a multicenter, hospital-based cohort, the findings may not be generalizable to populations eligible for mass CRC screening. Therefore, validation studies in broader, community-based populations are also warranted. Third, although fecal me*SDC2* testing may incur higher initial costs than FIT, its improved sensitivity for CRC detection could yield downstream clinical benefits through earlier diagnosis and treatment. Moreover, the conditional repeat-testing strategy employed in this study enhanced specificity, potentially reducing unnecessary colonoscopy referrals. A formal cost-effectiveness analysis was beyond the scope of the present study and should be addressed in future population-based implementation research. Fourth, another inherent limitation of stool DNA testing is the heterogeneous distribution of exfoliated tumor cells within stool specimens, which may result in false-negative findings if the collected portion contains insufficient tumor-derived DNA. To minimize this risk, participants were instructed to collect stool from multiple sites within the specimen and transfer it immediately into a preservative buffer [9,10]. Specimen adequacy was additionally verified using a human genomic DNA internal control, with inadequate samples retested according to the study protocol. Nevertheless, variability in stool sampling remains an inherent limitation of stool-based molecular assays and should be considered when interpreting test performance. Fifth, a further limitation of this study is that the potential influence of benign gastrointestinal conditions—including inflammatory bowel disease, chronic intestinal inflammation, diarrhea, constipation, and dietary and medication-related factors—was not systematically evaluated. Although prior analytical validation studies [9,10] demonstrated no significant interference from a broad range of commonly encountered medications and dietary substances, the effects of chronic inflammatory conditions on stool DNA methylation-based detection remain incompletely understood. Future prospective studies specifically designed to assess these potential confounding factors are warranted. Sixth, the present study did not evaluate cost-effectiveness, reimbursement status, or healthcare resource utilization. However, the assay can be performed using a standardized stool collection kit containing a DNA stabilization buffer validated for room-temperature storage for up to 30 days, facilitating specimen transport without specialized cold-chain requirements. Future studies should assess the economic impact, reimbursement considerations, and implementation feasibility of the assay in population-based screening programs. Finally, the study population comprised almost entirely of Korean participants; hence, validation in other racial and ethnic groups is necessary to confirm the broader applicability of the results.

## 5. Conclusions

The *SDC2* methylation test demonstrated high sensitivity and specificity for CRC detection, consistent performance across demographic and clinical subgroups, and modest positivity for AA, supporting its potential utility as a noninvasive screening tool for CRN. Furthermore, the performance of the test for CRC detection was consistently maintained from the regulatory approval phase through real-world clinical use, supporting its clinical validity, high reproducibility, and potential for broader clinical application.

## Figures and Tables

**Table 1:** Demographic characteristics of the study population.

	Total Population (*n* = 636)	Normal (*n* = 182)	Colorectal Polyp (*n* = 173)	Colorectal Cancer (*n* = 260)	Gastric Cancer (*n* = 11)	Liver Cancer (*n* = 10)
Age, mean (SD)	59.6 (10.5)	56.4 (10.5)	59.9 (8.7)	61.2 (10.6)	62.0 (10.7)	64.5 (6.3)
Age group, *n* (%)						
30–39	26 (4.1)	11 (6.0)	4 (2.3)	10 (3.8)	1 (9.1)	0 (0.0)
40–49	90 (14.1)	42 (23.1)	23 (13.3)	25 (9.6)	0 (0.0)	0 (0.0)
50–59	152 (23.9)	48 (26.4)	39 (22.5)	59 (22.7)	4 (36.4)	2 (20.0)
60–69	283 (44.5)	69 (37.9)	96 (55.5)	110 (22.7)	3 (27.3)	5 (50.0)
70–80	85 (13.4)	12 (6.6)	11 (6.4)	56 (21.5)	3 (27.3)	3 (30.0)
Sex, *n* (%)						
Male	322 (50.6)	71 (39.0)	100 (57.8)	135 (51.9)	7 (63.6)	9 (90.0)
Female	314 (49.4)	111 (61.0)	73 (42.2)	125 (48.1)	4 (36.4)	1 (10.0)

SD, standard deviation.

**Table 2:** Clinical performance of the me*SDC2* test for colorectal cancer using stool DNA.

Clinical Performance	Final Diagnosis		
	Colorectal Cancer	Normal	Total
Test results			
Positive	228	52	280
Negative	32	324	356
Total	260	376	636
Performance, % (95% CI)			
Sensitivity	87.7 (83.7–91.7)		
Specificity *			
Negative results on colonoscopy	91.8 (87.8–95.8)		
Non-CRC	86.2 (82.7–89.7)		
Accuracy	86.2 (84.2–89.4)		
Positive predictive value **	3.1 (2.4–4.0)		
Negative predictive value **	99.9 (99.9–100.0)		
False-positive rate	13.8 (10.3–17.3)		
False-negative rate	12.3 (8.3–16.3)		

* Non-CRC: colonoscopy-confirmed normal subjects, individuals with colorectal polyps, and patients with gastric and hepatic cancer. ** Positive and negative predictive values were calculated based on a colorectal cancer prevalence of 0.5%**,** which was used to determine the sample size.

**Table 3:** Clinical performance of the *SDC2* methylation test for colorectal cancer according to age group, sex, and year of testing.

	Number of Patients	Performance			
		Sensitivity, % (95% CI)	*p*-Value	Specificity, % (95% CI)	*p*-Value
Age groups			0.861		0.832
30–39	21	80.0 (55.2–100.0)		100.0 (100.0–100.0)	
40–49	67	92.0 (81.4–100.0)		90.5 (81.6–99.4)	
50–59	107	88.1 (79.9–96.4)		89.6 (80.9–98.2)	
60–69	179	86.4 (80.0–92.8)		92.8 (86.6–98.9)	
70–80	68	89.3 (81.2–97.4)		91.7 (76.0–100.0)	
Sex			0.885		0.935
Male	206	87.4 (81.8–93.0)		91.5 (85.1–98.0)	
Female	236	88.0 (82.3–93.7)		91.9 (86.8–97.0)	
Year of test			0.705		0.566
2023	233	88.8 (82.2–95.3)		92.4 (88.0–96.7)	
2024	209	87.1 (82.1–92.2)		89.5 (79.7–99.2)	

CI, confidence interval; *p*-values were tested using the chi-square test.

**Table 4:** Positivity rate of the *SDC2* methylation test for colorectal cancer according to tumor location and stage.

	Number of Patients	Positive *SDC2* Methylation Test		
		Number (%)	95% CI	*p*-Value
Location				0.798
Right-sided	68	59 (86.8)	78.7–94.8	
Left-sided	191	168 (88.0)	83.3–92.6	
Location by segments				0.186
Cecum	5	3 (60.0)	17.1–100.0	
Ascending colon	47	41 (87.2)	77.7–96.7	
Transverse colon	16	15 (93.8)	81.9–100.0	
Descending colon	8	5 (62.5)	29.0–96.1	
Sigmoid colon	109	99 (90.8)	85.4–96.2	
Rectosigmoid junction	7	6 (85.7)	59.8–100.0	
Rectum	67	58 (86.6)	78.4–94.7	
Both location	1	1 (100.0)	100.0–100.0	
TNM stages				0.671
Stage 0	14	12 (85.7)	67.4–100.0	
Stage I	50	41 (82.0)	71.4–92.7	
Stage II	56	49 (87.5)	78.8–96.2	
Stage III	94	84 (89.4)	83.1–95.6	
Stage IV	46	42 (91.3)	83.2–99.5	

CI, confidence interval; *p*-values were tested using the chi-square test.

**Table 5:** Comparative analysis of the clinical performance of the *SDC2* methylation test, fecal immunochemical test, and their combination in colorectal cancer.

	*SDC2* Methylation Test	FIT	Combination Test	* *p*-Value	** *p*-Value
^†^ Performance					
Sensitivity	87.1 (82.9–91.3)	81.0 (76.2–85.9)	95.6 (93.0–98.1)	0.045	<0.001
Specificity	86.2 (82.7–89.7)	94.3 (92.0–96.7)	82.4 (78.5–86.3)	<0.001	0.188
Sensitivity by stage					
Early (0–II) stage	96 (84.2)	83 (72.8)	106 (93.0)	0.037	0.038
Late (III–IV) stage	120 (89.6)	118 (88.1)	131 (97.8)	0.741	0.006

FIT: fecal immunochemical testing. * *p*-value indicates comparison between the me*SDC2* test and the FIT, and ** *p*-value indicates comparison between the me*SDC2* test and the combined test. ^†^ Performance data are expressed as % (95% confidence interval).

## Data Availability

The data can be obtained from the corresponding author upon reasonable request due to privacy restrictions.

## Supplementary Materials

The following supporting information can be downloaded at: https://www.mdpi.com/article/10.3390/diagnostics16121901/s1, Table S1: Positivity rate of the *SDC2* methylation test in non-CRC lesions.

## Abbreviations

AA	Advanced adenoma
CRC	Colorectal cancer
CRN	Colorectal neoplasia
C_T_	Cycle threshold
FDA	Food and Drug Administration
FIT	Fecal immunochemical test
NPV	Negative predictive value
PCR	Polymerase chain reaction
PMS	Post-market study
PPV	Positive predictive value
ROC	Receiver operating characteristic
SDC2	Syndecan-2
