# Supplementary material for: A Validation Study of Methylated Syndecan-2 in Stool DNA for the Detection of Colorectal Cancer
Source: Diagnostics (Basel). 2026 Jun 19;16(12):1901. doi: 10.3390/diagnostics16121901 (PMC13298951; doi:10.3390/diagnostics16121901)
Supplement: Supplementary file 1 [file diagnostics-16-01901-s001.zip › diagnostics-4350417-supplementary.pdf]

Supplementary Table S1. Positivity rate of the *SDC2* methylation test in non-CRC lesions.

Subgroups	Number of patients	Positive *SDC2* methylation test	
		Number (%)	95% CI
CRN (excluding CRC)	139	26 (18.7)	12.2–25.2
Advanced adenoma	46	13 (28.3)	15.3–41.3
Non-advanced adenoma	87	12 (13.8)	6.6–21.0
Serrated lesions	6	1 (16.7)	0.0–46.5
Non-CRN	34	6 (17.6)	4.8–30.5
Gastric cancer	11	3 (27.3)	1.0–53.6
Hepatic cancer	10	2 (20.0)	0.0–44.8

CRN, colorectal neoplasia; CRC, colorectal cancer; CI, confidence interval.

Supplementary Table S2. Stability of stool specimens stored at room temperature.

Samples	Days							
	0		10		20		37	
	COL2A1 C_T*	*SDC2* C_T*	*COL2A1* C_T*	*SDC2* C_T*	*COL2A1* C_T*	*SDC2* C_T*	*COL2A1* C_T*	*SDC2* C_T*
Negative-1	28.62 ± 0.45	ND	27.00 ± 0.11	ND	27.58 ± 0.31	ND	27.41 ± 0.45	ND
Negative-2	27.54 ± 0.18	ND	26.10 ± 0.10	ND	26.36 ± 0.24	ND	26.18 ± 0.14	ND
Negative-3	25.57 ± 0.08	ND	24.27 ± 0.12	ND	24.36 ± 0.11	ND	24.34 ± 0.06	ND
Negative-4	28.21 ± 0.37	ND	27.08 ± 0.03	ND	27.39 ± 0.20	ND	27.54 ± 0.18	ND
Negative-5	22.97 ± 0.41	ND	22.13 ± 0.05	ND	23.91 ± 0.24	ND	22.34 ± 0.17	ND
Negative-6	23.44 ± 0.12	ND	22.23 ± 0.04	ND	22.49 ± 0.13	ND	22.35 ± 0.06	ND
Positive-1	27.24 ± 0.32	31.19 ± 0.95	27.31 ± 0.39	31.96 ± 0.36	26.89 ± 0.07	30.77 ± 0.33	26.32 ± 0.16	30.13± 0.52
Positive-2	25.16 ± 0.15	29.98 ± 0.51	23.92 ± 0.48	29.62 ± 0.47	24.12 ± 0.03	29.91 ± 0.62	25.22 ± 0.30	30.04 ± 0.64
Positive-3	27.71 ± 0.45	32.17 ± 0.55	27.45 ± 0.07	32.39 ± 0.61	27.38 ± 0.09	32.31 ± 0.61	27.65 ± 0.04	32.44 ± 0.71
Positive-4	24.48 ± 0.08	27.58 ± 0.71	23.88 ± 0.03	26.99 ± 0.49	25.27 ± 0.37	27.41 ± 0.81	24.07 ± 0.19	27.64 ± 0.59
Positive-5	23.11 ± 0.16	30.16 ± 0.85	22.12 ± 0.03	30.64 ± 0.75	23.58 ± 0.18	31.11 ± 0.62	22.76 ± 0.04	30.91 ± 0.47
Positive-6	27.12 ± 0.19	28.19 ± 0.67	27.12 ± 0.34	29.01 ± 0.54	27.02 ± 0.04	29.14 ± 0.81	26.42 ± 0.14	28.76 ± 0.61

ND, not detected.

*Values are presented as mean ± standard deviation (SD) from three replicate measurements

[*] COL2A1 C_T values remained within the predefined acceptance criterion (baseline C_T ± 2.0) throughout 37 days of storage at 25–30°C. No changes in qualitative test results were observed, indicating that stool specimens collected in the collection kit were stable for at least 37 days under room-temperature conditions and support the established storage claim of 30 days.
